# Psychosis and homicide

**DOI:** 10.1192/j.eurpsy.2021.2110

**Published:** 2021-08-13

**Authors:** S. Kolsi, S. Hentati, I. Baati, J. Masmoudi

**Affiliations:** Psychiatry A, CHU Hédi Chaker Sfax, Sfax, Tunisia

**Keywords:** homicide-crime-violence-psychosis

## Abstract

**Introduction:**

Violence and crime committed by individuals with mental disorders has been the focus of growing interest among mental health professionals.Added to psychopathological disorders, individual, socio familial and therapeutic factors can be involved in the criminogenic risk.

**Objectives:**

To assess the characteristics of homicide in Tunisian patients with psychosis and to establish their sociodemographic, clinical and therapeutic characteristics.

**Methods:**

We reported7cases of patients who attended Psychiatric department“A”at the Hedi Chaker university hospital in Sfax,Tunisia,between January2014 and September2019.They were hospitalized for committing homicide and penal irresponsibility was recognized.

**Results:**

The homicide acts were matricide in 3cases, parricide in one case and conjugal homicide in one case.The homicide was not premeditated, committed by using knife weapon in 3cases and a blunt object in 4cases.The crime was done in the family home in the majority of cases(71.42%). The average age of patients was 34years. They were in almost cases(85.71%) male. Six patients (85.71%) had very low educational and income levels. They were mostly unmarried(71.42%)and unemployed(71.42%).Alcohol consumption was observed in3patients.However, we did not found any substance use. According to DSM-5,six patients were diagnosed with schizophrenia and one case with schizoaffective disorder.The majority(85.71%) had previous psychiatric folow-up. Furthermore, interruption of treatment was the rule.Five patients had a previous record of violent behavior towards the victim.Judicial history was notified among one patient.The persecution and influence delusion were found among 6cases.

**Conclusions:**

Homicidal behavior is extremely rare.Evaluation of different variables of homicide’s act and offender is a fundamental issue for developing preventive and therapeutic strategies to deal with such criminal behavior.

**Disclosure:**

No significant relationships.

